# Use of Botulinum Toxin A to Manage Hamstring-Induced Flexion Contracture Following Total Knee Arthroplasty: A Case Series

**DOI:** 10.7759/cureus.53113

**Published:** 2024-01-28

**Authors:** Quincy T Cheesman, Danielle Y Ponzio, Hope E Thalody, Vincent W Lau, Zachary D Post, Alvin Ong

**Affiliations:** 1 Orthopedic Surgery, Jefferson Health New Jersey, Stratford, USA; 2 Orthopedic Surgery, Rothman Orthopedic Institute, Egg Harbor Township, USA

**Keywords:** total knee arthroplasty, range of motion, flexion contracture, hamstring, botox, botulinum toxin a

## Abstract

Introduction

Flexion contractures following total knee arthroplasty (TKA) greatly affect patient function and satisfaction. Botulinum toxin A (BTX) has been described in the literature as a means of addressing post-operative flexion contractures due to hamstring muscle rigidity.

Methods

We retrospectively report a case series of eight patients with range of motion (ROM) who developed a flexion contracture status post-TKA and were managed with the use of physical therapy, diagnostic hamstring lidocaine injections, and therapeutic hamstring BTX injections.

Results

All patients had an improvement in extension ROM following diagnostic lidocaine hamstring injections and were therefore considered candidates for therapeutic hamstring BTX injections. Prior to therapeutic hamstring BTX injections, patients had an average flexion contracture of 19° (range: 15°-22°). All patients had an improvement in extension ROM two to four weeks following the therapeutic hamstring BTX injection, with an average improvement in ROM of 7° (range: 2°-19°). At the final follow-up, all patients continued to sustain an improvement in extension ROM with an average deficit of 9° (range: 0°-17°).

Conclusion

Our case series highlights the use of diagnostic hamstring lidocaine injections to confirm hamstring rigidity as an etiology for flexion contracture following TKA. In addition, we showed a persistent improvement in flexion contracture for all patients after hamstring BTX injections. Therefore, when the appropriate patient is selected, BTX may provide an additional treatment option for a flexion contracture following TKA.

## Introduction

Total knee arthroplasty (TKA) is highly effective in treating debilitating pain and improving the quality of life for patients with degenerative joint disease [1]. However, despite advancements in surgical technique, implant design, post-operative management, and pain control, post-operative stiffness continues to occur with a prevalence ranging from 1% to 20% [2-5]. Loss of range of motion (ROM) following TKA greatly affects function and satisfaction [6]. For example, patients may develop difficulty in performing activities of daily living (ADL) that leads to a decrease in mobility and independence. Furthermore, loss of ROM can lead to an altered gait pattern subsequently increasing patients’ risk for falls [7].

The incidence of a fixed flexion contracture following TKA ranges from 3% to 17% [6,8]. Eliminating pre-operative and intra-operative etiologies, it is thought that flexion contractures result due to a lack of adequate rehabilitation following surgery, lack of sufficient muscle tone, development of scar tissue, and muscle rigidity. The initial treatment modality for a flexion contracture within 12 weeks of TKA classically involves aggressive physical therapy aimed at improving the flexibility of scar tissue around the knee joint and enhancing the tone of the surrounding muscles [9]. If a patient lacks significant improvement in ROM despite vigorous physical therapy, manipulation under anesthesia (MUA) may be performed to manually disrupt adhesions contributing to decreased ROM [10]. Of importance, a MUA is recommended within 12 weeks of TKA in order to maximize the substantial gains in ROM and diminish the risk of complications [11-14].

The use of botulinum toxin A (BTX) has been described in the literature as a means of addressing post-operative flexion contractures due to hamstring muscle rigidity [15-17]. BTX is a neuromuscular blocking agent that acts selectively at peripheral cholinergic nerve endings by preventing the release of acetylcholine at the neuromuscular junction. This causes temporary paralysis, which reduces muscle rigidity and tone, allowing for improved control and balance across joints [15]. From an orthopedic standpoint, BTX is used to treat muscle spasms in patients with neurological disorders, such as Parkinson’s disease, cerebral palsy, and post-stroke spasticity [18]. However, the data on the use of BTX for post-operative TKA flexion contractures due to hamstring muscle rigidity is scarce. It is important to note that if the flexion contracture is due to other intrinsic or extrinsic causes, then hamstring rigidity may develop secondarily and can inhibit any improvement in extension. Therefore, BTX may be beneficial for achieving full extension even if the flexion contracture is not primarily due to hamstring rigidity [17].

We report a case series of eight patients who developed a flexion contracture status post-TKA and were managed with the use of physical therapy, diagnostic hamstring lidocaine injections [19], and therapeutic hamstring BTX injections. We highlight the use of a diagnostic hamstring lidocaine injection to confirm hamstring rigidity as an etiology of flexion contracture prior to the utilization of hamstring BTX injections. Additionally, we aim to contribute to the paucity of literature on BTX as a treatment modality for loss of ROM after TKA to help guide future research and clinical practice.

## Materials and methods

This study was a retrospective case series of eight patients who developed a flexion contracture status post-TKA and were managed by a board-certified, fellowship-trained orthopedic surgeon and a board-certified, fellowship-trained pain management and rehabilitation (PM&R) physician. Approval from our institution's Institutional Review Board (IRB) was obtained prior to the initiation of this study. The purpose of the study was to report ROM outcomes for patients with flexion contractures following TKA secondary to hamstring rigidity, which was confirmed with a diagnostic hamstring lidocaine injection and then subsequently treated with therapeutic hamstring BTX injections.

Inclusion criteria included patients who underwent primary TKA secondary to degenerative joint disease and subsequently developed a flexion contracture, which failed treatment with six weeks of aggressive physical therapy. Patients were subsequently diagnosed with hamstring rigidity, identified via diagnostic hamstring lidocaine injections, and treated with hamstring BTX injections. 

**Figure 1:**
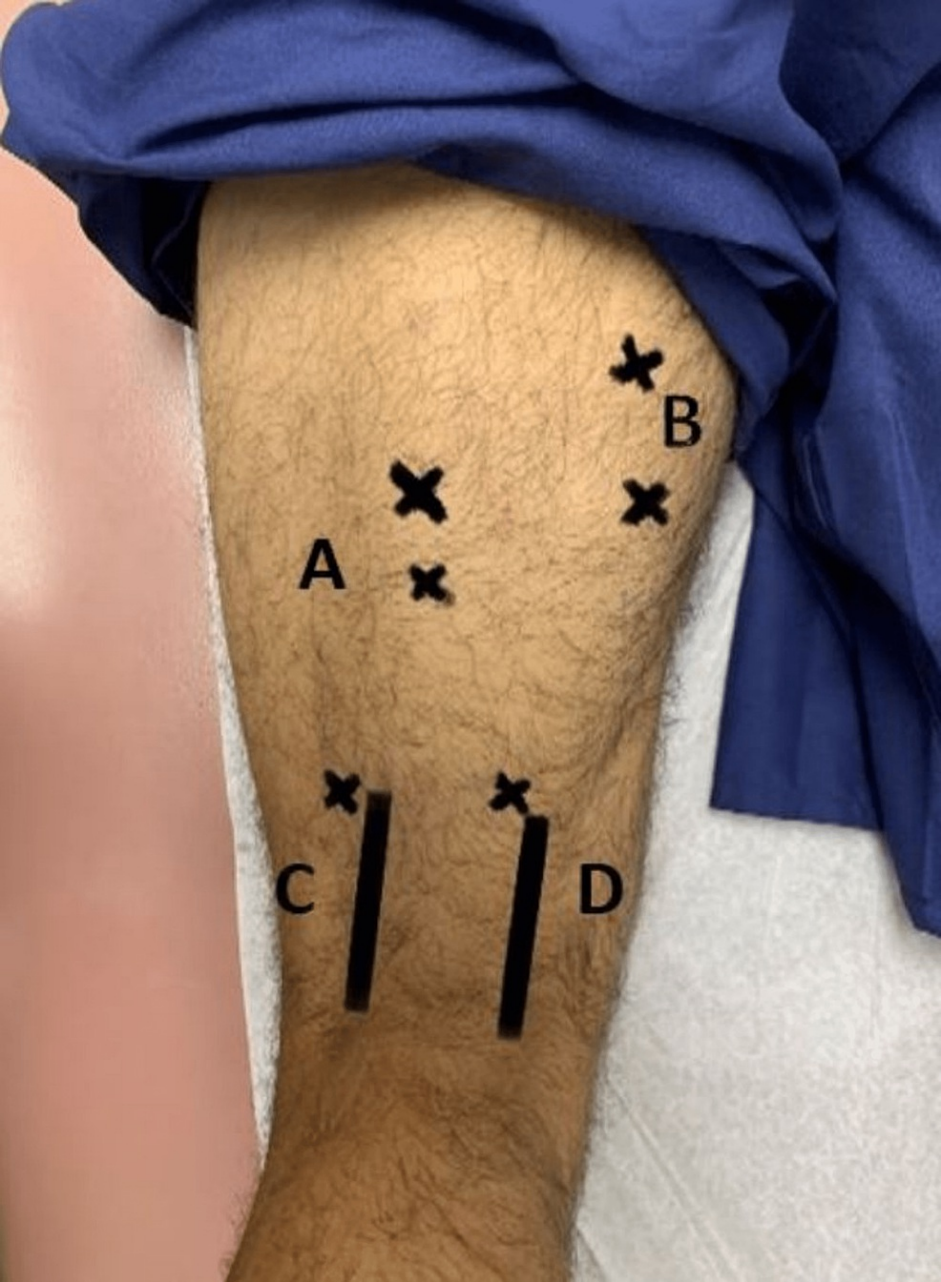
Location of hamstring injection sites: the patient is lying prone. (A) Long head biceps femoris; (B) semitendinosus; (C) short head biceps femoris; and (D) semimembranosus.

Of note, at our institution, all patients with continued flexion contractures at six weeks post-operatively are offered a MUA. If patients had a recurrent flexion contracture following a MUA or chose not to pursue a MUA, they were referred to a PM&R physician. At the first PM&R visit, roughly three to four weeks following MUA, a flexion contracture secondary to hamstring rigidity was confirmed. The PM&R physician utilized a goniometer to measure knee ROM. A lidocaine injection into the muscle belly of the four hamstrings (long head of biceps femoris, short head of biceps femoris, semitendonosis, and semimembranosis) was utilized to diagnose muscle rigidity. The patient was placed prone on the exam table, and an injection of 1.0-1.5cc of 1% lidocaine, using a 25-gauge needle, was placed into each muscle belly of the four hamstrings. To determine the location of injection, the long head of the biceps femoris and the semitendinosus muscle belly were palpated in the region of the mid-thigh, while the short head of the biceps femoris and semimembranous muscle belly were located by measuring approximately 8-10 cm proximally from the inferior tendinous portion of the muscle (Figure 1). If there was an improvement in knee extension following the injection, this confirmed hamstring rigidity as a contributing factor to the flexion contracture, and the decision was made to proceed with BTX injection therapy one to four weeks later as a means of sustained improvement.

The BTX injection was performed under electromyography (EMG) guidance. BTX was reconstituted with 1 mL of 0.9% normal saline per 100 units of BTX powder prior to injection. The patient was placed in a prone position, and EMG was utilized to guide the injection into each of the four muscle bellies of the hamstring. Varying units of BTX were injected into each hamstring muscle based on the patient’s BMI. Patients then continued aggressive physical therapy plus home exercises until follow-up in the orthopedic surgeon’s clinic, approximately two to four weeks post-BTX injections.

## Results

Eight patients were included in the present study (Table 1). The mean age was 66 years old (range: 43-75). The median time to diagnosis of flexion contracture by a PM&R physician was 11 weeks post-operative from TKA. Of the eight patients, only four underwent a MUA. The median post-operative time to treatment with therapeutic hamstring BTX injections was 14 weeks.

**Table 1:** Right knee range of motion throughout the post-operative course. TKA: total knee arthroplasty, MUA: manipulation under anesthesia, ROM: range of motion, R: right, L: left, M: male, F: female, N/A: not applicable.

TKA	Age/sex	Laterality	Date of TKA	Pre-MUA ROM	Post-MUA ROM	Pre-lidocaine ROM	Post-lidocaine ROM		Pre-botox ROM	Area of botox injection and units of botox	Post-botox ROM	Post-botox ROM
TKA’s with manipulation under anesthesia followed by hamstring botox injections												
1	74/M	R	10/19/2020	12/04/2020 10°-90°	12/04/2020 0°-120°	12/23/2020 20°-95°	12/23/2020 15°-95°	01/11/2021 15°-110°		Biceps femoris long 120, Biceps femoris short 80, Semitendinosus 120, Semimembranosus 80	01/26/2021 10°-110°	03/23/2021 7°-105°
2	73/M	L	10/05/2020	11/23/2020 15°-120°	11/23/2020 0°-130°	12/14/2020 15°-90°	12/14/2020 5°-90°	01/06/2021 20°-105°		Biceps femoris long 100, Biceps femoris short 100, semitendinosus 100, semimembranosus 100	02/04/2021 10°-110°	03/04/2021 6°-110°
3	69/M	R	10/26/2020	12/11/2020 10°-75°	12/11/2020 0°-120°	12/15/2020 15°-75°	12/15/2020 10°-85°	02/03/2021 20°-100°		Biceps femoris long 120, Biceps femoris short 80, Semitendinosus 120, Semimembranosus 80	02/24/2021 12°-100°	10/11/2022 0°-120°
4	66/F	R	04/19/2021	06/07/2021 10°-90°	06/07/2021 0°-120°	07/07/2021 21°-90°	07/07/2021 13°-98°	07/28/2021 19°-93°		Biceps femoris long 120, Biceps femoris short 80, Semitendinosus 120, Semimembranosus 80	08/19/2021 0°-110°	10/26/2021 0°-115°
TKA’s with hamstring botox injections only												
5	60/F	L	07/08/2020	N/A	N/A	06/29/2021 20°-85°	06/29/2021 10°-95°	07/27/2021 18°-93°		Biceps femoris long 120, Biceps femoris short 80, Semitendinosus 120, Semimembranosus 80	08/12/2021 16°-90°	11/16/2021 15°-90°
6	65/F	L	12/16/2020	N/A	N/A	02/17/2021 25°-80°	02/17/2021 18°-85°	03/09/2021 22°-80°		Biceps femoris long 100, Biceps femoris short 100, Semitendinosus 100, Semimembranosus 100	03/22/2021 16°-80°	05/24/2021 13°-80°
7	75/F	L	12/14/2020	N/A	N/A	04/28/2021 23°-110°	04/28/2021 15°-110°	05/24/2021 19°-110°		Biceps femoris long 100, Biceps femoris short 100, Semitendinosus 100, Semimembranosus 100	06/15/2021 15°-110°	09/21/2021 10°-110°
8	43/F	L	10/29/2021	N/A	N/A	04/29/2022 20°-110°	04/29/2022 12°-110°	05/18/2022 20°-105°		Biceps femoris long 80, Biceps femoris short 70, Semitendinosus 80, Semimembranosus 70	06/08/2022 15°-115°	07/06/2022 17°-115°

The average flexion contracture was 20° (range: 15°-25°) prior to the diagnostic lidocaine hamstring injections. All patients had an improvement in extension ROM following the diagnostic lidocaine hamstring injection, with the average improvement in ROM of 8° (range: 5°-10°). Prior to therapeutic hamstring BTX injections, patients had an average flexion contracture of 19° (range: 15°-22°). All patients had an improvement in extension ROM two to four weeks following the therapeutic hamstring BTX injection, with an average improvement in ROM of 7° (range: 2°-19°). At the final follow-up, a median time of 13 weeks from therapeutic hamstring BTX injection, all patients continued to sustain an improvement in extension ROM with an average deficit of 9° (range: 0°-17°).

## Discussion

A flexion contracture following a TKA can have a significant effect on patient functionality leading to challenges in post-operative mobility. There are limited treatment modalities available after a flexion contracture is identified. These include aggressive physical therapy, MUA, and arthroscopic lysis of adhesion. This case series describes an additional treatment modality with the use of diagnostic lidocaine injections to identify hamstring rigidity, followed by therapeutic hamstring BTX injections.

A review of the literature regarding the use of BTX for flexion contractures following TKA has revealed promising results. Seyler et al. followed 11 TKA patients for two years with an initial flexion contracture of >10°. Patients were managed with BTX injections in the hamstring muscles if they had complaints of hamstring tightness in the setting of a clinical examination demonstrating a loss of complete knee extension. At two-year follow-up, nine of the 11 patients reached 10° to 0° of extension. Furthermore, they found almost all patients had good to excellent clinical outcomes based on the Knee Society Score [15].

In addition, Smith et al. performed a randomized controlled trial assessing 15 patients with flexion contractures greater than 10° at one month post-operative. Patients were randomized to either a BTX cohort or a placebo cohort. Patients’ hamstring muscles were injected with BTX or saline, and ROM follow-up was performed at one-, six-, and 12-month intervals. The BTX cohort had improved extension ROMs at each interval, with an average extension of 8.0°, 5.1°, and 1.1°, respectively. The saline placebo cohort had improved extension ROMs at each interval, with an average extension of 3.8°, 2.2°, and 1.3°, respectively. Mixed model regression reported a one-year post-injection extension improvement of 18° (±7.5°) for the BTX cohort and 12° (±2°) for the placebo cohort (p=0.04). However, both the BTX cohort and placebo cohort reached within 1-2° of full extension at one-year follow-up. This calls into question the clinical significance of BTX since both groups reached similar outcomes in regard to extension ROM [17].

Candidate selection for BTX is not uniform and varies among studies. Seyler et al. used a combination of clinical examination ROM, muscle strength, leg length discrepancy, and video gait analysis to select eligible candidates. Smith et al. only utilized clinical examination ROM with a flexion contracture greater than 10° to select eligible candidates. In our study, we utilized a diagnostic trigger point injection with lidocaine to determine hamstring rigidity as a contributing factor of flexion contractures; thus, a candidate for BTX. 

Despite the majority of patients demonstrating improvements in flexion contracture over time, it is important to note that not everyone improved to an extension ROM less than 5°. Patients with a continued flexion contracture expend more energy in their quadriceps to help load bear through the knee and maintain stability, leading to quick fatigue when standing, walking, and climbing stairs. Thus, the ideal extension angle is between 0° and 5° to prevent the progression of the flexion contracture and to avoid the aforementioned complications [20]. The lack of full extension in some of our patients may be due to concomitant arthrofibrosis or other multifactorial etiology; therefore, they were unable to be fully corrected by a hamstring BTX injection.

## Conclusions

Given the paucity of literature regarding this treatment modality, further studies and research will continue to be important to assist in proper patient selection and treatment technique. Our case series showed an improvement in flexion contracture for the majority of patients. Therefore, when the appropriate patient is selected, BTX may provide an additional treatment option for flexion contractures following TKA.
